# COVID-19 Pandemic Consequences among Individuals with Eating Disorders on a Clinical Sample in Poland—A Cross-Sectional Study

**DOI:** 10.3390/ijerph19148484

**Published:** 2022-07-11

**Authors:** Przemysław Seweryn Kasiak, Natalia Adamczyk, Alicja Monika Jodczyk, Aleksandra Kaproń, Anna Lisowska, Artur Mamcarz, Daniel Śliż

**Affiliations:** 1 Students’ Scientific Group of Lifestyle Medicine, 3rd Department of Internal Medicine and Cardiology, Medical University of Warsaw, 04-749 Warsaw, Poland; przemyslaw.kasiak1@gmail.com (P.S.K.); adamczyk.natalia1997@gmail.com (N.A.); kapron98@gmail.com (A.K.); a.lisowskaaa@gmail.com (A.L.); daniel.sliz@wum.edu.pl (D.Ś.); 2 Polish Society of Lifestyle Medicine, 00-388 Warsaw, Poland; artur.mamcarz@wum.edu.pl; 3 3rd Department of Internal Medicine and Cardiology, Medical University of Warsaw, 04-749 Warsaw, Poland; 4 School of Public Health, Postgraduate Medical Education Centre, 01-813 Warsaw, Poland

**Keywords:** eating disorder, COVID-19 pandemic, mental health, social media, symptoms worsening, coping strategies, depression, lifestyle changes

## Abstract

The COVID-19 pandemic and imposed restrictions had negative consequences on overall health among many populations. This study aimed to investigate the influence of the pandemic on eating disorders (ED) and mental health (MH) of individuals with confirmed ED diagnoses. A survey consisting of questions related to (1) diagnosis of COVID-19, (2) changes in ED symptoms and onset of new symptoms, (3) psychological and MH aspects regarding to the pandemic, (4) lifestyle changes, and (5) social media (SM) usage was distributed between April–June 2021. One hundred and ninety-eight individuals met all of the inclusion criteria (*n*_females_ = 195, 98.48%; *n*_other gender_ = 3, 1.52%). Of the participants, 78.79% reported worsening of their ED symptoms, 42.93% of them noticed an onset of new ED symptoms, and 57.58% believed that the pandemic had a negative impact on their ED treatment. Negative changes due to the pandemic on MH were reported by 88.89%. Of the participants, 91.92% increased their time spent on SM and 54.04% of them declared that it had a negative impact on their MH. Medical professionals should consider results while providing comprehensive psychological care, which can be crucial information in the application of the appropriate treatment strategy.

## 1. Introduction

The COVID-19 pandemic has influenced the lifestyles of people around the world, disrupting the functioning of social, economic, and healthcare systems [1]. In Poland, the epidemic was announced by the government on the 20 March 2020 [2] and abolished on the 16 May 2022 [3]. The implemented restrictions, such as quarantine periods, closure of numerous institutions, and social distancing policies, have resulted in detrimental mental health consequences globally. The mental health post-isolation problems include, among others, depression, anxiety, mood disorders, low self-esteem, lack of self-control, and other adverse mental health (MH) outcomes [4].

This harmful influence seems to have had a particular effect on individuals with, or at risk of, eating disorders (ED) [5]. There have not been any large-scale population studies on the subject of ED prevalence and the impact of the pandemic carried out in Poland. However, reports across the world have shown an increased incidence of ED behaviors and diagnoses, but also the deterioration of ED in patients already diagnosed, including exacerbation of ED symptoms and comorbidities in the COVID-19 pandemic [5]. Taquet et al. [6] observed an increase in the incidence of ED by 15.3% in 2020, compared to the years before the pandemic. The steady increase in the relative risk of ED in women and girls was also demonstrated from March 2020, primarily only concerning adolescents and anorexia nervosa (AN) [6].

ED are serious, potentially fatal, and high-cost mental disorders that exacerbate physical health and disrupt psychosocial functioning [7]. During the COVID-19 pandemic, ED symptoms could be exacerbated by the disruption to daily activities, routine and structure, modification in physical activity and sleep habits, social isolation, media exposure and increased screen time, fear of food shortages, and fear of contagion [8,9]. Moreover, in confinement times, access to protective sources for patients, including social support, adaptive coping strategies, and treatment, is limited [8]. The limitations on doing grocery shopping may have various influences on eating behaviors. They may affect the development of restrictions on calorie intake and skipping meals but, on the other hand, may also increase binge eating as a result of food insecurity, and hoarding food at home [9,10].

This study aimed to investigate the impact of the prolonged COVID-19 pandemic and lockdowns on patients with the diagnosis of ED, such as anorexia nervosa, bulimia nervosa (BN), atypical anorexia nervosa (AAN), binge eating disorder (BED), and other specified or non-specified ED in Poland. We hypothesized that the pandemic had an adverse influence on individuals with ED by the exacerbation of symptoms, changes in the quality and quantity of consumed food, and disrupting ongoing treatment.

## 2. Materials and Methods

### 2.1. Survey Instrument

The self-report survey was directed to individuals suffering from varied ED. It was distributed among patients from the Dialogue Therapy Centre (www.terapiadialog.pl (accessed on 6 April 2022), Warsaw, Poland) and additionally with online methods. The Dialogue Therapy Centre patients were recruited during their visits (during the visit, informed consent was obtained to participate in the study prior to completing and submitting the survey). With the online methods, the questionnaire was exported to groups on social media websites where individuals with ED were present and active. The password, known only by individuals in the groups, and informed consent were obtained on the first page of the online survey. The survey was anonymous, and this information was mentioned at the beginning of the questionnaire.

### 2.2. Eligibility Criteria

**Figure 1:**
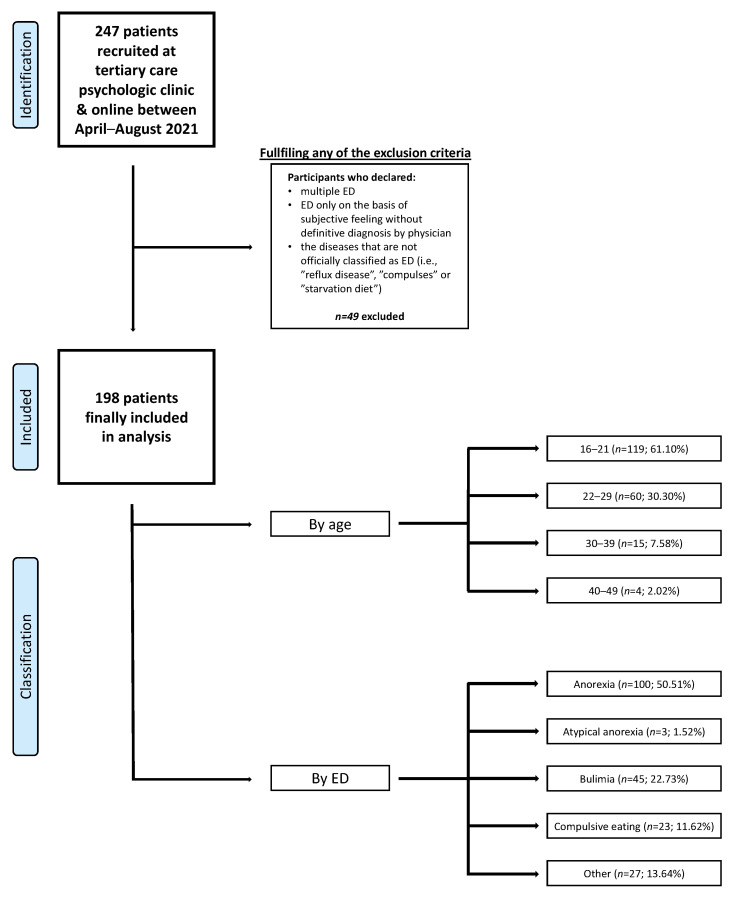
Flowchart of the participants selection process. Study population consisted of patients who declared themselves as females (*n* = 195; 98.48%) or did not want to specify gender (*n* = 3; 1.52%). There was no males willing to participate in the study. Data are presented as the number of patients and as a percentage of the whole group. Age classification has been shown in years. Abbreviations: ED, eating disorder.

The survey was conducted between the 7 April and the 12 June 2021. It was at the time of the peak of the third wave of the pandemic in Poland, the implementation of multiple restrictions, and the beginning of the vaccination program available to everyone. To qualify for the study, participants had to meet two main preliminary criteria: (1) being diagnosed with an ED and (2) currently undergoing treatment. The minimum age to participate was 16 years. Participation in the study was fully voluntary. Participants did not receive any form of gratification for completing the questionnaire. To eliminate the impact of the unviable answers and enrich data validity, a data cleaning process was implemented. Participants who indicated that they simultaneously had an exceptive diagnosis, e.g., bulimia nervosa (BN) and anorexia nervosa (AN) or AN and binge eating disorder (BED), were excluded due to our suspicion that they did not have awareness of their diagnosis or their diagnosis varied during their lifetime [11]. Answers which suggested that the person did not have a diagnosis, such as “I do not have a diagnosis”, “starving”, or “eating bouts”, were excluded. Figure 1 presents a flow diagram of the whole participants selection process.

The survey contained the authors’ questions based on the questionnaire from the study of Schlegl et al. [12], which was translated to Polish, the official language. The original questionnaire was created by a team of clinicians, psychotherapists, and researchers (the authors did not provide additional validation data). Our survey consisted of 3 main areas: (1) sociodemographic data: age, gender, diagnosed ED, living and occupational situation, and questions regarding the history of COVID-19 infection and health consequences of the pandemic on ED (worsening and/or self-perceived symptom onset of the ED, impact on the treatment of ED, and changes in specific symptoms and eating-related behaviors); (2) changes in eating habits (i.e., quality and quantity of certain foods) and physical activity; and (3) lifestyle and general psychopathology changes (quality of life, mental health, and general psychopathology symptoms, such as sadness, loneliness, helplessness, etc.) and social media (SM) usage. The full survey, consisting of questions assigned to each section, is available in the Supplementary Materials (S1-Questionnaire).

### 2.3. Data Analysis

Basic data was saved into an Excel file (Microsoft Corporation, Washington, DC, USA). Further, the data were calculated as mean (±standard deviation; SD) and median. Normality was tested with the usage of the Shapiro–Wilk test. Differences between an overall number of responses (the continuous variables) were calculated with the usage of the one-way ANOVA test and the post-hoc Tukey’s HSD test (for parametric data) or Kruskal–Wallis test (for non-parametric data). To assess correlations between additionally selected variables, a Chi^2^ test was performed.

Statistical analyses were conducted in SPSS software (version 28; IBM SPSS, Chicago, IL, USA) and STATA software (Stata Corp, College Station, TX, USA; version 15.1). A two-sided *p*-value of <0.05 was considered as a benchmark of significance.

## 3. Results

### 3.1. Characteristics of the Respondents

A total number of 248 participants completed the form, of which 198 (79.84%) of them met the study conditions. The sample consisted of 195 females (98.48%) and 3 people who did not want to specify their gender (1.52%). There were no males participating in the study (no one completed the questionnaire). The mean age was 21.72 ± 9.00 years and the median was 22 years. Of our participants, 50.51% (*n* = 100) declared having AN, 22.73% (*n* = 45) BN, 11.62% (*n* = 23) BED, 1.52% (*n* = 3) atypical anorexia nervosa (AAN), and 13.64% (*n* = 27) other specified or non-specified ED. Table 1 shows the sample description. The majority of the participants lived with family or a partner (88.89%; *n* = 176) and were working/learning online (70.71%; *n* = 140). Of the participants, 74.75% (*n* = 148) reported a previous or past SARS-CoV-2 infection.

### 3.2. Impact of the COVID-19 Pandemic on Eating Disorders Symptoms and General Psychopathology Symptoms

Of the participants, 78.79% (*n* = 148) agreed that the pandemic resulted in worsening of their ED symptoms, 14.14% (*n* = 28) disagreed with that statement, and 7.07% (*n* = 14) had no opinion. Of the participants, 42.93% (*n* = 85) reported a self-perceived symptom onset of the ED, 38.89% (*n* = 77) did not notice any changes, and 18.18% (*n* = 36) had no opinion. A negative impact of the pandemic on the treatment of ED was reported by 57.58% (*n* = 114) participants, a positive influence by 13.13% (*n* = 26), no impact by 13.64% (*n* = 27), and 15.66% (*n* = 31) did not know. Table 2 shows the impact of the COVID-19 pandemic on ED symptoms and general psychopathology symptoms.

### 3.3. Differences between the Overall Number of Responses in Each Question

A Kruskal–Wallis test shows that the COVID-19 pandemic significantly influenced ED symptoms and eating behaviors, H (4) = 17.92, *p* = 0.001. The participants often significantly chose answers “Worsening” (median = 99.00) than “Hard to say” (median = 16.00) or “Not concerning” (median = 11.00). Table 3 presents *p*-values for each possible combination between answers about ED symptoms and eating behaviors.

A one-way ANOVA revealed that there was a significant difference in the number of responses related to the impact of the COVID-19 pandemic on self-being, F (df = 4 between groups; df = 40 within groups) = 177.71, *p* < 0.00001. The Tukey’s HSD test for multiple comparisons found that the mean number of “Worsening” (mean = 148.22 ± 28.22) answers were significantly higher than the number of all remaining answers (each *p* = 0.0001). There was also a significant difference between the number of participants who chose “Improvement” (mean = 7.00 ± 2.82) and “No change” (mean = 28.22 ± 15.34); *p* = 0.02, “Hard to say” (mean = 7.22 ± 5.33) or “No change” (mean = 28.22 ± 15.34); *p* = 0.02, and “No change” (mean = 28.22 ± 15.34) or “Not concerning” (mean = 4.00 ± 3.08); *p* = 0.01. Table 4 presents *p*-values for each possible combination between answers about self-being.

### 3.4. Impact of the COVID-19 Pandemic on Quality of Life and Lifestyle

Of the respondents, 81.82% (*n* = 162) claimed that the pandemic caused a general deterioration in the quality of their life, 6.57% (*n* = 13) did not notice any changes, and 5.56% (*n* = 11) did not know. Negative changes in mental health were reported by 88.89% (*n* = 176) of the participants, positive changes were reported in 3.54% (*n* = 7), 4.04% (*n* = 8) did not see any changes, and 3.54% (*n* = 7) had no opinion. The limitation on the possibilities of meeting with other people negatively influenced the symptoms of ED of 66.67% (*n* = 132) respondents, positively influenced 11.62% (*n* = 23), did not influence 13.64% (*n* = 27), and 8.08% (*n* = 16) did not know.

Among individuals with AN, 62% ate less food than before the pandemic, 22% ate more, 8% did not see any changes, and 8% did not have an opinion. Among individuals with BN, 55.56% ate more than before the pandemic, 31.11% ate less, 2.22% did not see any changes, and 11.11% did not know. Among individuals with BED, 86.96% ate more, 4.35% ate less, 4.35% did not see any changes, and 4.35% did not know.

Among participants with AN, 54% exercised more than before the pandemic, 39% less, and 7% did not see any changes. Among those suffering from BN, 33.33% exercised more, 60% less, and 6.67% did not see any changes. Among individuals with BED, 73.91% exercised more and 26.09% exercised less.

### 3.5. COVID-19 Pandemic Consequences Resulted from SM Usage on ED

Of the participants, 91.92% (*n* = 182) increased their SM usage during the pandemic, 6.06% (*n* = 12) decreased usage, and 2.02% (*n* = 4) did not see any changes. Among people who increased time spent on SM, 54.04% (*n* = 107) saw their negative impact on ED symptoms, 16.67% (*n* = 33) saw a positive impact, 9.09% (*n* = 18) did not noticed an impact, and 14.14% (*n* = 28) did not have an opinion.

### 3.6. Correlations

The pandemic’s impact on mental health was correlated with higher usage of SM (*p* = 0.02; OR = 6.3; CI = 1.11–35.7). However, there was no significant correlation between the pandemic’s impact on mental health and self-perceived symptom onset of ED (*p* = 0.89; OR = 1.13; CI = 0.22–7.76), changes in PA (*p* = 0.41; OR = 0.49; CI = 0.09–2.74), or living alone (*p* = 0.15; OR = 4; CI = 0.51–31.08). COVID-19 infection was not correlated with the onset of new symptoms of ED (*p* = 0.13; OR = 2.3; CI = 0.76–7.03).

### 3.7. Open Questions

Our survey also included open questions in particular sections. Descriptive answers obtained from each individual are included in the Supplementary Materials (S2-Answers in open questions). Briefly, respondents mainly reported the negative impact of the COVID-19 pandemic, indicating the worsening of their ED symptoms, a reduction in the amount of physical activity, and expressed their fear and uncertainty about the future. The responses varied in narration and length, from single words to multi-sentence statements. A few of the respondents indicated a positive effect, arguing it provided more time for themselves to take care of their well-being, ED treatment, and mental health.

## 4. Discussion

The novelty of our study is its pioneering nature. To the best of our knowledge, this is the first study on Polish individuals with ED, which characterizes the impact of the COVID-19 pandemic on ED symptoms, mental health, ongoing treatment, and social media use. This is the first research conducted on an ED clinical sample indicating the consequences of a long-term pandemic influence. It has the potential to draw the attention of clinicians and caregivers and raise awareness of the problem locally in Eastern Europe and worldwide. It may influence future health guidance and policies for patients with ED after the pandemic, considering the extent of the problem.

We describe the impact of the COVID-19 pandemic on individuals with ED at the time of the peak of the third wave of the pandemic in Poland, the implementation of multiple restrictions, and the beginning of the vaccination program available to everyone. Therefore, our results indicate the consequences of recurring lockdowns and prolonged social limitations and may highlight long-lasting and future issues in the field of eating disorders. Moreover, during data collection, the majority of our respondents reported former or current COVID-19 infection, which might have influenced the results.

ED concern mostly females and are very uncommon in males, thus, the lack of gender-specific results in our study can be explained [13]. Moreover, ED may be underdiagnosed and undertreated among men, which has an impact on the research in this field [14]. Interestingly, ED and body dissatisfaction are common in sexual gender minorities and a lifetime prevalence of AN, BN, and BED is higher in transgender individuals than in cisgender heterosexual individuals, concerning mostly transgender men [15]. In our study, we did not collect data from individuals from the sexual minority population, however, some of them chose not to specify their gender while completing the survey.

The majority of our respondents claimed worsening of ED symptoms as the result of the pandemic, mainly including fear of gaining weight, body dissatisfaction, and drive for thinness. This is in line with other research showing ED symptoms deterioration in a variety of population groups [16,17,18]. Over 50% of our respondents noticed worsening in restrictive eating, 50% in binge eating, and a significant number of individuals also noticed the onset of new symptoms. In contrary to our results, Termorshuizen et al. [19] found that only over one third of participants in the United States and the Netherlands reported worsening of dietary restriction and compensatory behaviors. However, that study was focused on the early impact of the pandemic on ED symptoms in contrast to the recurring and prolonged restrictions in ours. What is important is in our study, individuals with AN ate less during the pandemic than before, while patients with BN and BED ate more. It indicates the differences between these groups and may implicate the exacerbation of specific eating disorder symptoms, such as caloric restrictions in AN and binge eating in BN and BED. Many of our respondents also claimed that limitation on meeting with other people negatively influenced their symptoms. It emphasizes the role of social support in maintaining the well-being and recovery of patients with ED found in other studies [8]. Moreover, as available in the Supplementary Materials (S2-Answers in open questions) from answers to open questions, for some of the respondents, the confinement caused the relapse of their disorder or the diagnosis of ED for the first time, which was also observed in other studies and shows the potential of ED extent after the pandemic [5].

A disruptive influence on the ED treatment was reported by over half of our respondents. This result is in line with many previous studies showing a percentage of 28–74% of individuals noticed the adverse impact of the pandemic on their treatment [12,18,20,21]. It could be caused by limitations in access to in-person therapy and difficulties in transitioning and adapting to telehealth services. Although online medical encounters seem to be comparable to traditional encounters and are safe, tolerated, and effective for improving ED symptoms and mental health [16,22,23], other studies’ results found that only less than 10% of patients were willing to remain in online treatment after the confinement [21,24].

It is already known that the COVID-19 pandemic has caused a deterioration in mental health among many populations [25,26,27,28]. It contributed to the appearance and exacerbation of depression, anxiety, stress, psychological distress, post-traumatic stress disorder, and sleep disturbances (especially insomnia), among others [27,28,29,30]. Moreover, the pandemic caused the exacerbation of various ED, including increasing the tendency of orthorexia nervosa, which greatly worsened the mental health of individuals with these disorders [31,32]. Most of our respondents (over 80%) reported that the pandemic caused a general deterioration in the quality of their life. Negative changes in mental health were reported by nearly 90% of them, while positive changes were only reported by 3.5%. The majority of the participants reported a worsening in feeling: sadness, being overwhelmed, helplessness, fear for the future, and loneliness. The majority reduced their self-assessment. Individuals with ED have, in general, a low tolerance for uncertainty [33]. They tend to have lower acceptance of emotions, and less emotional awareness and clarity [34]. They demonstrate significantly higher levels of emotional intensity and have an increased use of functional emotion regulation strategies [34]. The exposure to stress caused by the COVID-19 pandemic could have contributed to the increase in anxiety and depression symptoms among our participants.

The influence of SM on the mental health of individuals has already been investigated before the pandemic [35,36]. The studies have revealed ambiguous relationships [36]. The same ambivalence has been found in the context of the current pandemic [35]. Although SM may be used as a source of information and entertainment or for communication with others, the research shows that a higher amount of time spent on SM is correlated with poorer mental health [37]. Several studies have revealed that the media promote an unrealistic ideal of beauty and that can be the reason for body dissatisfaction, especially among adolescents [38,39,40]. Our study showed that almost all participants spent more time on SM than before the pandemic. More than half of them believed that it contributed to their poorer mental health, while only 16% perceived a positive impact. Based on previous research, it can be assumed that the perception of how one’s illness is portrayed in the media can have consequences for the self-perception, well-being, and actions of ill persons [12,38].

Several limitations should be considered when interpreting the results. Firstly, the sample is relatively small, and more cross-sectional research on wider populations should be performed. Thus, our results should be interpreted carefully. Although our survey did not have gender preferences, we did not succeed in collecting data from men. It can be partially explained by the population data for the prevalence of ED (which mostly affects females). Because the data were self-reported, they potentially could be biased. Some of our participants were recruited online, and we cannot be sure that they fully understood the study protocol. 

## 5. Conclusions

The results of this study indicate the magnitude of deterioration in ED symptoms during the COVID-19 pandemic and suggest the possibility of increased help-seeking of individuals with ED after the pandemic. Individuals noted the harmful consequences on their ED of aggravating symptoms, lowering diet quality, deterioration in mental health, and disrupting the ongoing treatment. Raising awareness of the problem in Poland and worldwide is crucial to provide comprehensive care for individuals with ED and to apply an appropriate treatment strategy.

## Figures and Tables

**Table 1:** Demographic characteristics of the participants.

	Number/Percentage
**Gender**	
**Female**	*n* = 195 98.48%
**Male**	*n* = 0 0.00%
**Not specified**	*n* = 3 1.52%
**Age**	
**16–21**	*n* = 119 61%
**22–29**	*n* = 60 30.3%
**30–39**	*n* = 15 7.53%
**40–49**	*n* = 4 2.02%
**ED**	
**AN**	*n* = 100 50.51%
**BN**	*n* = 45 22.73%
**BED**	*n* = 235 11.62%
**AAN**	*n* = 3 1.52%
**Other**	*n* = 27 13.64%
**Place of living**	
**Rural**	*n* = 48 24.24%
**Town < 50 thousands of inhabitants**	*n* = 38 19.19%
**Town 50–100 thousand inhabitants**	*n* = 25 12.63%
**City >100 thousand inhabitants**	*n* = 87 43.94%
**Living situation during pandemic**	
**Alone**	*n* = 22 11.11%
**With partner/family**	*n* = 176 88.89%
**Online learning/working**	
**Yes**	*n* = 140 70.71%
**No**	*n* = 30 15.15%
**Partially**	*n* = 28 14.14%
**SARS-CoV-2 infection (confirmed by a test)**	
**Yes**	*n* = 148 74.75%
**No**	*n* = 50 25.25%

Data are presented as numbers and percentages. Age is presented in years. Abbreviations: ED, eating disorder; AN, anorexia nervosa; BN, bulimia nervosa; BED, binge eating disorder; AAN, atypical anorexia nervosa; SARS-CoV-2, severe acute respiratory syndrome coronavirus 2.

**Table 2:** Pandemic impact on ED symptoms and general psychopathology symptoms.

How Have Your ED Symptoms and Eating Behaviors Changed during the COVID-19 Pandemic?					
	Number of Participants/Percentages				
	Worsening	Improvement	No Change	Hard to Say	Not Concerning
**Fear of gaining weight**	*n* = 161 81.32%	*n* = 7 3.54%	*n* = 12 6.06%	*n* = 16 8.08%	*n* = 2 1.01%
**Drive for thinness**	*n* = 128 64.65%	*n* = 18 9.09%	*n* = 33 16.67%	*n* = 16 8.08%	*n* = 3 1.52%
**Body dissatisfaction**	*n* = 137 69.19%	*n* = 13 6.57%	*n* = 33 16.67%	*n* = 10 5.05%	*n* = 5 2.53%
**Limitation of the amount or the frequency of the meals**	*n* = 117 59.09%	*n* = 28 14.14%	*n* = 25 12.63%	*n* = 18 9.09%	*n* = 10 5.05%
**Restrictive eating**	*n* = 103 52.02%	*n* = 28 14.14%	*n* = 34 17.17%	*n* = 22 11.11%	*n* = 11 5.56%
**Binge eating**	*n* = 99 50%	*n* = 20 10.1%	*n* = 20 10.1%	*n* = 17 8.59%	*n* = 42 21.21%
**Self-induced vomiting**	*n* = 56 28.28%	*n* = 16 8.08%	*n* = 29 14.65%	*n* = 3 1.52%	*n* = 94 47.47%
**Laxatives abuse**	*n* = 26 13.13%	*n* = 15 7.58%	*n* = 31 15.66%	*n* = 10 5.05%	*n* = 116 58.59%
**Diuretics abuse**	*n* = 11 5.56%	*n* = 15 7.58%	*n* = 35 17.68%	*n* = 5 2.53%	*n* = 132 66.67%
**Snacking/unplanned eating**	*n* = 96 48.48%	*n* = 120 10.1%	*n* = 23 11.62%	*n* = 19 9.6%	*n* = 40 20.2%
**Appetite**	*n* = 87 43.94%	*n* = 50 25.25%	*n* = 27 13.64%	*n* = 25 12.63%	*n* = 9 4.55%
**How Did the COVID-19 Pandemic Influence Your Self-Being?**					
	**Number of Participants/Percentages (%)**				
	**Worsening**	**Improvement**	**No Change**	**Hard to Say**	**Not Concerning**
**Loneliness**	*n* = 152 76.77%	*n* = 6 2.53%	*n* = 28 14.14%	*n* = 9 4.55%	*n* = 3 1.52%
**Fear for the future**	*n* = 159 80.3%	*n* = 5 2.53%	*n* = 22 11.11%	*n* = 8 4.04%	*n* = 4 2.02%
**Fear for one’s health**	*n* = 110 55.56%	*n* = 8 4.04%	*n* = 57 28.79%	*n* = 15 7.58%	*n* = 8 4.04%
**Sadness**	*n* = 181 91.41%	*n* = 5 2.53%	*n* = 8 4.04%	*n* = 2 1.01%	*n* = 2 1.01%
**Reduced self-assessment**	*n* = 153 77.27%	*n* = 8 4.04%	*n* = 29 14.65%	*n* = 5 2.53%	*n* = 3 1.52%
**Sleep disturbances**	*n* = 111 56.06%	*n* = 13 6.57%	*n* = 48 24.24%	*n* = 16 8.08%	*n* = 10 5.05%
**Loss of control of one’s life**	*n* = 154 77.78%	*n* = 9 4.55%	*n* = 26 13.13%	*n* = 5 2.53%	*n* = 4 2.02%
**Helplessness**	*n* = 170 85.86%	*n* = 4 2.02%	*n* = 19 9.6%	*n* = 4 2.02%	*n* = 1 0.51%
**Overwhelming**	*n* = 174 87.88%	*n* = 5 2.53%	*n* = 17 8.59%	*n* = 1 0.51%	*n* = 1 0.51%

Data are presented as numbers and percentages. Abbreviations: ED, eating disorder; COVID-19, coronavirus disease 19.

**Table 3:** Differences between an overall number of respondents for each answer in questions related to ED symptoms and eating behaviors changes due to the COVID-19 pandemic.

Type of Answer	Worsening	Improvement	No Change	Hard to Say	Not Concerning
**Worsening**	–	0.06	0.67	**0.0006**	**0.04**
**Improvement**	0.06	–	1.00	1.00	1.00
**No change**	0.67	1.00	–	0.30	1.00
**Hard to say**	**0.0006**	1.00	0.30	–	1.00
**Not concerning**	**0.04**	1.00	1.00	1.00	–

Kruskal–Wallis test was performed to assess intergroup differences. Significant values (*p* < 0.05) were bolded.

**Table 4:** Differences between the overall number of respondents for each answer in questions related to the impact of the COVID-19 pandemic on self-being.

Type of Answer	Worsening	Improvement	No Change	Hard to Say	Not Concerning
**Worsening**	–	**0.0001**	**0.0001**	**0.0001**	**0.0001**
**Improvement**	**0.0001**	–	**0.02**	1.00	0.99
**No change**	**0.0001**	**0.02**	–	**0.02**	**0.01**
**Hard to say**	**0.0001**	1.00	**0.02**	–	0.99
**Not concerning**	**0.0001**	0.99	**0.01**	0.99	–

One-way ANOVA and post-hoc Tukey’s HSD test were performed to assess intergroup differences. Significant values (*p* < 0.05) were bolded.

## Data Availability

The data presented in this study are available on request from the corresponding author. The data are not publicly available due to not obtaining consent from respondents to publish the data.

## Supplementary Materials

The following supporting information can be downloaded at: https://www.mdpi.com/article/10.3390/ijerph19148484/s1, S1: Questionnaire form, S2: Answers in open questions.
