# Supplementary material for: COVID-19 Pandemic Consequences among Individuals with Eating Disorders on a Clinical Sample in Poland—A Cross-Sectional Study
Source: Int J Environ Res Public Health. 2022 Jul 11;19(14):8484. doi: 10.3390/ijerph19148484 (PMC9321998; doi:10.3390/ijerph19148484)
Supplement: Supplementary file 1 [file ijerph-19-08484-s001.zip › ijerph-1781578-supplementary.pdf]

Supplementary material S1- Questionnaire form

We invite you to complete the following survey on the impact of the COVID-19 pandemic on eating disorders. The survey is aimed at people diagnosed with an eating disorder undergoing treatment. The minimum age for participation in the study is 16 years.

Period of the COVID-19 pandemic: March 20, 2020 - present day

The average time to complete the survey is 5 minutes.

The survey is anonymous and its results will be used for research purposes.

Thank you for your time.

Section 1.- general and demographic questions

1. What is your gender?

_____ (female / male / I would rather not to give it)

2. What is your height?

_____ (in cm)

3. What is your weight?

_____ (in kg)

4. What is your age?

_____ (in years)

5. What is your place of living?

_____ (city <50 000 residents / city 50 000-100 000 residents / city >100 000 residents / village or countryside)

6. What eating disorder have you been diagnosed with?

_____ (anorexia nervosa / bulimia nervosa / binge-eating disorder / other- participants could wrote their own answer)

7. Who are you living with during the COVID-19 pandemic?

_____ (alone / with a partner or with family)

8. Are you studying / working from home?

_____ (yes / no)

9. Have you been or are you currently infected with SARS-COV-2 confirmed with a positively test result?

_____ (yes / no)

Section 2.- questions related to COVID-19 pandemic on eating habits, mental health, and lifestyle

1. Has the COVID-19 pandemic worsened your eating disorder symptoms?

_____ (yes / no / I don't know)

2. Did you develop new eating disorder symptoms during the COVID-19 pandemic?

_____ (yes / no / I don't know)

3. If there were new symptoms, what are they?

_____ (open question- participants wrote answer)

4. Has the COVID-19 pandemic worsened your quality of life?

_____ (yes / no / I don't know)

5. Has the pandemic affected the amount of food you eat?

_____ (yes- I eat more food / yes- I eat less food / no / I don't know)

6. Has the pandemic affected the quality of the food you eat?

_____ (yes- I eat higher-quality products / yes- I eat lower-quality products / no / I don't know)

7. Has the COVID-19 pandemic affected the treatment of your eating disorder?

_____ (yes- influenced positively / yes- influenced negatively / no / I don't know)

8. Has limited opportunities to meet other people during COVID-19 pandemic affected your eating disorder?

_____ (yes- influenced positively / yes- influenced negatively / no / I don't know)

9. Has the COVID-19 pandemic affected your mental health?

_____ (yes- influenced positively / yes- influenced negatively / no / I don't know)

10. How has the COVID-19 pandemic affected your physical activity?

_____ (I exercise or move slightly less/ I exercise or move much less/ I exercise or move slightly more/ I exercise or move much more / no)

11. Is the pandemic associated with your increased use of social media?

_____ (yes / no / I don't know)

12. If the answer to the previous question was yes, is social media information influencing your eating disorder?

_____ (yes- influenced positively / yes- influenced negatively / no / I don't know)

13. How have your symptoms and behaviors related to each of the following conditions changed during the COVID-19 pandemic? [multipoint question; answer was the same for each point]

_____ (Worsening / Improvement / No change / Hard to say / Not concerning)

- Fear of gaining weight
- Drive for thinness
- Body dissatisfaction
- Limitation of the amount or the frequency of the meals
- Restrictive eating
- Binge eating
- Self-induced vomiting
- Laxatives abuse

- Diuretics abuse
- Snacking / unplanned eating
- Appetite

14. How has the COVID-19 pandemic affected each of the following life habits? [multipoint question; the answer was the same for each point]

_____ (Worsening / Improvement / No change / Hard to say / Not concerning)

- Loneliness
- Fear for the future
- Fear for one's health
- Sadness
- Reduced self-assessment
- Sleep disturbances
- Loss of control of one's life
- Helplessness
- Overwhelming

15. We encourage you to share your personal experiences and thoughts about the impact of the COVID-19 pandemic on eating disorders.

_____ (open question- participants wrote answer)

Supplementary material S2- Answers in open questions

Answers in question 3 [part 2]:

“I get irritated when dinner is not on time, for example.”

“Slowing down the metabolic rate.”

“Binge eating.”

“Obsessed with taking steps.”

“Vomiting.”

“Compulsive behaviors.”

“Vomiting, fasting.”

“Less control, eating huge amounts of food, feeling unwell with myself.”

“All accompanying anorexia, excessive exercise, food restriction, weight loss, depressive states, mood swings, etc.”

“Attempted vomiting, depression, obsessive exercise.”

“Fear of gaining weight (again), fear of eating an "excess" amount of food, fear of providing any calories beyond fulfillment of the basic metabolic rate.”

“Compulsive sports.”

“Obsessive exercise.”

“Binge eating, sports, bulimia.”

“All.”

“Additional steps.”

“In fact, during the pandemic, I fell into an eating disorder, when the media began to promote being fit and exercise a lot, so as not to gain weight during the pandemic.”

“Obsession with burning kcal.”

“All slimming started.”

“Establishing and sticking to fixed meal times.”

“Starvation.”

“No appetite, change of favorite dishes.”

“Increased control because my food depended only on me (not on the hours of classes, what I can take away from home, etc.).”

“During the first wave of the pandemic, I fell into anorexia.”

“I fell into anorexia.”

“Addiction to sports (burning kcal).”

“I am more obsessed with kcal. I have also suffered from laxative bulimia from sports bulimia itself.”

“More disgust for the body, greater possibility of binge eating because I'm at home more often, photophobia.”

“Planned binge eating.”

“Hitherto unknown to me scale of compulsive eating attacks.”

“Excessive exercise.”

“Implementation of fasts, the emergence of greater bouts of eating, obsession with steps and exercise.”

“More exercise and more binge eating.”

“Total closing in on myself, I do not want to talk to anyone, cut off from the world. From the physical symptoms, an even greater decline in strength. In addition, I started having panic attacks (fast heartbeat).”

“Provoking vomiting.”

“More frequent retching.”

“It was hard for me to eat something that is not semi-liquid.”

“Trials to vomit / vomiting.”

“Obsession with being active, saving calories, fear of eating out.”

“In addition to vomiting, I started taking laxatives.”

“Obsessive thoughts about food.”

“Intense physical exertion.”

“Addiction to specific unhealthy foods, habit of eating to pass the time, stress eating.”

“Eating out of "boredom", mindless snacking.”

“I devoted much more time to training, practically complete isolation from others, and earlier it was not possible when there was a school because I had to attend it.”

“The fear of eating has increased.”

“Not eating for a few days.”

“Reluctance to eat, obsessive focus on my appearance, starvation.”

“I started starving, exercising more, obsessively counting everything I ate and drank.”

“Bulimic symptoms”

“In general, I induced vomiting and prayed for a lean body, exercised at night, and there was not an hour when I did not measure my hands.”

“Fear of eating something with an unknown number of calories.”

“Worsening of heart problems.”

“Greater selectivity of products.”

“Also bulimia.”

“I have the impression that I eat even more mainly to improve my mood.”

“Fear of eating, intense physical activity (I started to have wounds on my body), fear of gaining weight, amenorrhea, hair loss.”

“Suicidal thoughts, orthorexia, calorie counting, body measurement.”

“All accompanying anorexia, excessive exercise, food restriction, weight loss, depressive states, mood swings, etc.”

“I started to weigh everything I ate again, increased my physical activity (even one hour interval training + 20k steps, yt exercises for stomach, legs, arms), skipping meals, obsessive thoughts about eating "multiplied". During covid, I was hospitalized twice because of my low body weight.”

“Compulsive obsessive disorder.”

“All-day fasts, compulsive exercises, recently binge eating.”

“Fear of eating and binge eating; the number of binge eating and vomiting daily has increased; starvation, weight loss, symptoms of anorexia.”

“Reducing the size of meals to the smallest they can be.”

“The symptoms of gluttony came back.”

“The anorexia started.”

“Suicidal thoughts, orthorexia, calorie counting, body measurement.”

“Overeating.”

“Greater selectivity of products.”

“Last year, forcing myself to exercise, bouts of bulimia, tightening of caloric restrictions.”

“New fear foods, new strict rules for specific meals, more exercise.”

“Increased need for control, increased movement, fear of gaining weight.”

“Weighing all products, counting calories, depressed mood.”

“It was during the pandemic that the whole disorder began.”

“Compulsive overeating.”

“During the pandemic, I developed a general eating disorder.”

“Eating restriction.”

“Fear of gaining weight, excessive overeating.”

“Eating at night.”

“The pandemic caused the disorder.”

“More frequent binge eating.”

“Anorexia in general developed during the pandemic (started shortly before March 2020).”

Answers in question 15 [part 2]:

“Being at home helped me. When I was in my flat at university, I ate "cheap", that is little, and at home I ate more because there was more food and it made me recover a bit as I think”

"Remote learning is the biggest mistake that the authorities have treated us for over a year."

"The pandemic gave me the opportunity to spend more time with myself, which made me reflect on my fears, feelings, and the mechanisms of the eating disorders that I live with. It can be said that at least I "found the time" and was ready to make a conscious decision to seek help from a psychologist. The improvement of my mental state (including the relationship with the body) and the reduction of vomiting and binge eating results from the work I have done on myself. So, the pandemic by itself hasn't made it better. Instead, it created an environment conducive to my self-development. Calmness, less stress, and better organization of life had a positive effect on my approach to nutrition. There were also new difficulties with nutrition, I became aware of many sad facts and beliefs about myself that I had in my subconscious, which strongly affect my behavior and self-esteem. So far, the pandemic time has been fruitful for me. I have had many successes, although there are many stumbles and falls on this path. For me, the pandemic standstill is what I needed."

"The pandemic has revealed that my struggle has been going on for over 15 years. My body is no longer an anorexic, but my head is still there. I'm in therapy and I started it before the pandemic in 2019. Currently, I am focusing on solving these problems and regaining the freedom that the disease took from me when I was a teenager."

"Isolation can have a negative impact on people who need to gain weight because they are afraid to return to school with a changed body shape."

"I came out of anorexia, if you can call it that, almost 3 years ago. My weight was then 68 kg, I felt very comfortable in my body. I went to the gym, which gave me great satisfaction. With the pandemic, intrusive thoughts returned to me, I lost 6 kg, I do not feel well with myself. On the one hand, I would like to go back to what I looked like. On the other hand, I am afraid that I will gain too much weight. I eat irregularly now, not very healthy, but I'm trying to change that. I work out at home maybe once or twice a week. I don't like it very much because during advanced eating disorders I did it every day, so I have not very good memories. I have no motivation for anything, I feel very depressed. I have started to compare myself to others a lot again, and that is also having a bad effect on me."

"It was during the first lockdown that I got into an eating disorder. The internet and television promotes very much weight loss and exercise so as not to gain weight during a pandemic when we do not move so much. Reading false information on "fit" weight loss. Celebrities who actually promote orthorexia more than healthy eating. I started to exercise a lot and eat less, observing all these fitness accounts, and thus I made the worst decision of my life, starting to count calories and before I knew it, I lost 10 kg in just two months. Menstruation, mental health and I fell into the trap of a disease."

"During the pandemic, I had to rethink my life, and after 8 years of fruitless disruption and reluctance to get out of it, I decided to get out of it. The eating disorder was largely overcome, but the depression definitely got worse and the feeling of being unable to do

anything useful which is related to remote learning and returning to the family home for financial reasons.”

“My way of improving was the gym. During the treatment of disorders, I used the gym and it helped me, I had the comfort that I would exercise and I could eat, because it would even out, I would not gain weight, and I was also losing weight. Since the gyms are closed, I'm very bad.”

“At some point I felt that it was an ideal option to go back and listen to your body, go "all in" using limited meetings with friends, hence less fear of seeing that I have gained weight, or less fear of irritation with some texts, mentioning food, I just have more comfort of treatment when nothing "bothers me".”

“It is very difficult to lose weight, practically impossible working from home.”

“More time at home means more time to exercise, to separate myself from the people I have always received support from.”

“Initially, it was just my idea of healthy weight loss, but I took advantage of being at home isolated from others and started reducing calories to an absolute minimum to strive for perfection.”

"In my case, the pandemic and partial isolation had a positive effect on the recovery process, although I was in the most difficult period of treatment before the pandemic. The pandemic means that I do not compare to others as much as I used to, and the greater amount of free time allows me to again learn how to eat normally and look for physical activity that will give me pleasure. In social media I started to surround myself with content about recovery and body positivity, which is also helpful. Although there are very triggering voices from the media like "why are we getting fat in quarantine, how not to gain weight in a pandemic”, etc., it sends a signal that gaining weight is something negative.”

“I'm on recovery and it's getting better but still not as it should.”

“The longer the pandemic lasts, the longer it will be and the more people with eating disorders there will be.”

“In fact, it is during this sick isolation and being alone with yourself that you realize more that you are sick, and some people you thought were friends who also told you that they would be a support ... then suddenly you come to the conclusion that if you can count, you have to count on yourself and take care of yourself, because in the end you will be left alone with yourself, so it's better to be alone but healthy.”

“The lack of a daily routine, for example going to universities, worsened my mental state a lot. I feel like I'm wasting my time, I can't organize my routine. Lockdown does not help either, and returning to a small town with no interesting places only causes more resentment. I go to Instagram a lot and see these pretty smiling girls and I think to myself that I am an unattractive loser sitting at home who has not done anything interesting in

his life. I am locked at home, and they, dressed nicely, travel despite the virus and have fun as if nothing had happened. On Facebook, I see friends from high school, my age, who have husbands, wives, normal lives, pictures like that don't help either. More than normal, I brood over the past, regretting all the things I didn't do, all the years I had wasted. I have no motivation for anything, I don't want to study, read books, or paint for me, even though these are the things I have always liked to do, as if it made no sense at all. I could just lie on the bed staring out the window. I am in a long-distance relationship, and because of the restrictions, I never know when I will be able to visit my boyfriend, often you cannot buy a ticket due to the reduced number of places and you have to plan your trip earlier than before. Sometimes I have the feeling that I will always be unhappy. I stopped watching the news completely so as not to get more excited about the pandemic. I look forward to the decline in infections. When my grade is eligible for vaccination, I'll do it so we can go back to normal. Young medical students hold on and good luck <3."

"Humans are social creatures, so isolation from others is not natural. Currently my BMI is not life threatening, but during the pandemic my health has deteriorated significantly and all problems with eating disorders have returned with doubled strength. I was threatened with a psychiatric hospital, which I think, if not for the pandemic, I could have avoided relapse and control the disease, because the pandemic intensified everything (including depression). I know a lot of people with an almost identical situation. Isolation affects people badly. Some fall into eating disorders, others to self-mutilation or other forms of self-destructive behavior, because they cannot cope with this situation."

"I'm worried about my parents' earnings, I don't think I deserve to buy new things, including food. I eat monotonously."

"By having more free time, people spend more time online, where it's not hard to come across weight loss content, TikTok's to romanticizing eating disorders, or toxic recovery accounts. In the past, when my head was busier with school and friends, I treated this content more neutrally, that it was just next to me, it did not affect me as much as it does now. Now I have the feeling that I'm stuck in this bubble of food, physical activity, comparing myself. I think it is a bit due to the lack of spontaneity in my life, that I am in my comfort zone and can control my calorie consumption, activity and even noticed that I control my loved ones in terms of their food quality and quantity and their physical activity."

"My whole life during that first "quarantine" revolved around food, I was fed up."

"Personally, the pandemic period "helped" me fight my eating disorder, and I had more time to focus on recovering from the disease. On the other hand, the mental state was disturbed by the lack of contact with people and the lack of normality, but in my case, the current mental state does not exacerbate the eating disorders."

"Some of my answers may seem inconsistent with the overall answer, but that's because I'm in therapy. That is why "depression" and so on I marked "not applicable" because thanks to working with a therapist I feel much better in OVERALL. A social media only makes me feel better because I started following safe, neutral body profiles on IG.

However, I think that social isolation can promote weight gain. Because nobody is watching us."

"The pandemic gave me a lot of time to think and reflect on myself. I realized that I needed help and stopped ignoring and normalizing my symptoms. I started therapy, but the winter spent on remote learning made my sick thoughts intensify, because not having the opportunity to go out with friends, develop or enjoy my life, I felt that everything was losing sense, which also caused my motivation to fight anorexia somewhere was disappearing. Moments before starting therapy, my BMI was so low that I barely avoided hospitalization."

"I don't think the pandemic will have a negative impact on my eating disorder. I had more time for myself. I was able to focus on my needs. I was able to spend more time with my partner."

"I started to be afraid of eating in front of people again."

"It seems to me that by constantly weighing and checking in the mirror if I have gained weight, I am trying to fill a void and distract from other problems, i.e., loneliness and lack of will to live."

"The closure meant that if I go out (and I do it for some stability) I either go to work or to a store where I stock up large supplies, which I immediately devour as soon as I enter home. Inability to go to college (I study daily, I work part time) causes a decrease in physical activity, a shift in the hours of sleep (from 22/23- 7/8 to 1/2 at night- 9-10), and eating more food of inferior quality. The pandemic negatively impacted my relationship and ultimately led to breaking up, abandoning by a good friend, my mental health deteriorated. I had no contact with a psychiatrist since autumn, and from the beginning of the pandemic until the fall this contact was significantly worse (teleporting instead of visiting)."

"I eat my meals more often by myself, which means I eat them faster and have less control of what I eat. There is more highly processed food in my diet, and I also eat less - 3 (was 5) meals, which makes me feel guilty. Because of I go out "to people" less, I feel worse mentally, I have much lower self-esteem, which translates into eating under the influence of emotions - especially sweets."

"All my symptoms definitely got worse. I have been struggling with an eating disorder for 6 years and have had better and worse episodes of the disease. COVID-19 definitely caused me to fall back into restrictions and deteriorate my health. However, I still fight because I know that life without disease is much more beautiful."

"Nothing makes me happy; I don't see the point; every day is the same as in prison."

"I can't say much, but mainly because of the virus, I had the biggest relapse."

"From the beginning of the pandemic, I started to lose weight. The content I came across on social media significantly worsened my self-esteem and deepened my self-

acceptance problems, pushed me into disturbed behavior. Fortunately, I received help and support quickly, and now I'm in the process of recovering from an eating disorder, but isolation and studying at home don't make the process any easier."

"In my case, I miss my friends and we don't know when it will all end, and it overwhelms me."

"But now I have more time to prepare my meals."

"On the one hand, the worst moment of my disorders was a few months ago during the 2nd lockdown, but on the other hand, during the 2nd lockdown, I also gained a lot of weight and started recovery. Loneliness and the coming summer, however, overwhelms me so much that I again fall into bad habits, which I have never even tried to eradicate (even in recovery I practiced burning what I eat and skipped meals - despite everything the balance was positive, you can see it on weight)."

"The pandemic made me drown in my own fears and sorrows. It made depression, suicidal thoughts and bulimia my friends."

"I think I had a bad relationship with food before the pandemic, but it didn't affect my life that much, I didn't actually notice the problem. I was busy and had something to do. Today, sitting at home, being out of touch with people and boredom have caused binge eating. As for the media influencing disorders - I think there is more and more valuable content, e.g., on Instagram."

"I feel very lonely."

"The deterioration of my general mental state brought back my toxic relationship with food."

"For me, it looked like that before the pandemic, I was practically healthy after 2 years of recovering from it ... I kept the correct weight for 1.5 years, developed, and ate as much as I needed. Although already a difficult period for me was already starting and I noticed more snacking at home, for example. During this first stage of the pandemic (i.e., in 2020) it intensified even more - the more I ate, I had less movement. In May, I left the house of my own maybe a few times because I was so depressed. It affected my figure, I gained 2-3 kg on weight, but it influenced my self-esteem - I started to see it as a colossal difference, I was afraid how others would see me after so long. And this, along with other private matters that have not worked out for a long time, unfortunately propelled a return to the restrictions. As during the first part of the pandemic I was overeating, I did not control too much how much I eat (I ate healthy after all, but too much), so in 2 parts, i.e., from September to now, I started to starve again after 2.5 years, to fall into restrictions, obsessions, I lost quite a lot. It turned out that before the pandemic I felt almost free and I worked out a lot, I followed this common sense which, even in the moment of some small bad thoughts in the back of my head, drowned them out, so now, after a long time of this pandemic, I lost my motivation completely, I even tell myself that I don't want to go back to that moment, I don't even feel any reasons to recover, anorexia and orthorexia became such comfort, control."

“At the beginning of the pandemic, I started exercising and for the first time in my life I lost weight healthily, i.e., exercised a lot, but also ate healthy and was in a healthy deficit. With time, I noticed that this is becoming my obsession again, and even though I am not hungry, I keep an eye on my calories and feel compelled to exercise. When I came back to college after the holidays, it was only worse, which combined with my break up with my boyfriend. Since November, I've been obsessing over calories, eating less than I should, and thinking too much about eating.”

“The pandemic brought my eating disorders to light, but at the same time it caused me to start psychological and dietary treatment.”

“Due to the pandemic, I reached the apogee of my disturbed behavior and a slow decline. Now I have gained weight, partially released from the diet culture, regained menstruation. So, it can be said that the pandemic contributed to the deterioration and, as a result, improvement (although I am still in the process of recovery) of health.”

“I also have an avoidant personality diagnosis, so I'm glad that the pandemic exists - it gives me an excuse to cut myself off from the real world.”

“I'm a loner, so the pandemic is good for me, I don't like meeting people, so I'm glad we don't have to gather.”

“I believe that without the pandemic, I would not have fallen into bulimia.”

"Covid has a terrible effect on ed because there is no live therapy, there is no option to distance yourself from problems outside the home."

"I think that the pandemic is not conducive to overcoming eating disorders, remote work at home keeps me thinking" I have to go to exercise because I do not move enough "forcing myself to exercise despite the lack of strength and time due to school duties. My condition, the psychological condition has deteriorated enormously and it is also related to a certain private situation, currently I go to a psychologist because without it I could not cope with my life.

Escaping into psychoactive substances that ended very badly.

In a word, I think that without the pandemic, my life would be much better and my mental state would be in very good condition as it was before the pandemic. "

"In my case, there were big changes during the year: first, the symptoms worsened significantly, because I had more time to focus on eating: preparing meals myself, controlling how they are prepared at home, eating at fixed times, more time to plan meals and count. At some point I started noticing that something was wrong, I had a few conversations and started to strive for a change, then I would leave the house less often, I was not afraid of gaining weight necessary to fight the disorder and I had time to work through my problems with myself. So, it's hard for me to say for sure how the pandemic affected the disorder. "

"The pandemic started everything, everyone started to" be fit "with it, which of course is No. 1 in social media and thus the comparison started much more."

"The pandemic mainly increased my compulsive overeating. But it reduced the frequency of vomiting because it is more difficult to hide it when you are the whole time with your partner at home. It causes a huge psychological burden, weight gain and lower self-esteem."

"Due to the quarantine, I became obsessed with calories and food. It was partly due to observing fit Instagram girls that prompted me to get into the SUMMER form and not to improve my eating habits."

"When I sit at home all day at school, I have no strength and motivation for anything, I feel bad and my eating disorders are the only kind of sense that keeps my emotions in check, so if I did not starve myself, I would feel pissed that I cannot go anywhere and it would be a worse feeling that I was partially deprived of my freedom, and so I feel apathy and lack of strength due to anorexia and I do not want to go out and that is fine. "

“Deterioration.”

“The pandemic exacerbates or begins an eating disorder in many.”

“Forced isolation leaves a lot of space in life for nurturing anorexia and more time to think about eating / not eating, limiting daily activities (studying at home, I do not go anywhere) intensifies remorse occurring during / after eating.”
